# Supplementary material for: Compressed sensing based approach identifies modular neural circuitry driving learned pathogen avoidance
Source: bioRxiv. 2024 Oct 17:2024.04.10.588911. Preprint. [Version 2] doi: 10.1101/2024.04.10.588911 (PMC11507717; doi:10.1101/2024.04.10.588911)
Supplement: Supplement 4 [file NIHPP2024.04.10.588911v2-supplement-4.pdf]

bioRxiv preprint doi: https://doi.org/10.1101/2024.04.10.588911; this version posted October 17, 2024. The copyright holder for this preprint (which was not certified by peer review) is the author/funder, who has granted bioRxiv a license to display the preprint in perpetuity. It is made available under aCC-BY 4.0 International license.

Supplementary Materials

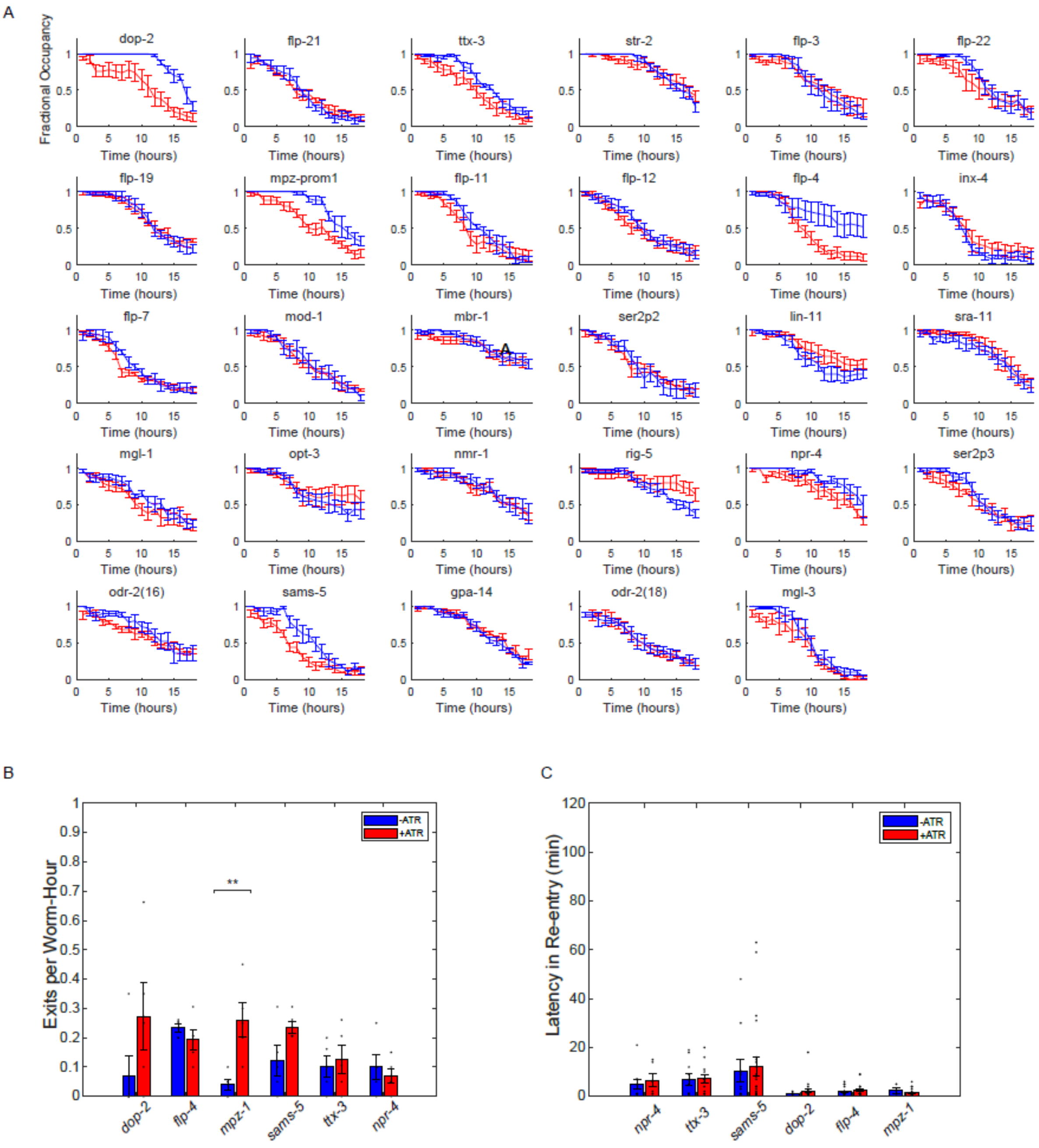

Fig.S1. Lawn evacuation following neural inhibition.

(A).Evacuation dynamics of all 29 transgenic lines following 2 hours of neural inhibition. **(B)**. Exit rate of worms on OP50 with neural inhibition. **(C)**. Latency of re-entry with neural inhibition.

bioRxiv preprint doi: https://doi.org/10.1101/2024.04.10.588911; this version posted October 17, 2024. The copyright holder for this preprint (which was not certified by peer review) is the author/funder, who has granted bioRxiv a license to display the preprint in perpetuity. It is made available under aCC-BY 4.0 International license.

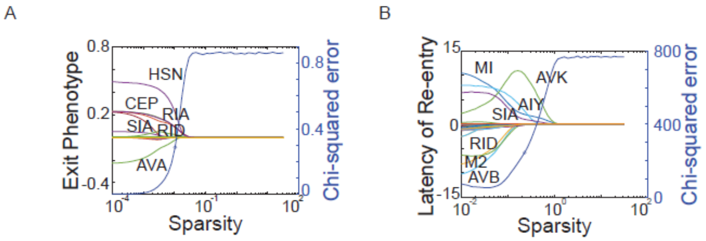

Fig.S2. Compressed sensing solutions with sparsity parameters for lawn exit and entry

(A) Median neuron weights from 10,000 lasso regression solutions over three orders of magnitude of sparsity parameters for lawn exit rate. **(B)** Median neuron weights from 10,000 lasso regression solutions over three orders of magnitude of sparsity parameters for lawn entry timescale.

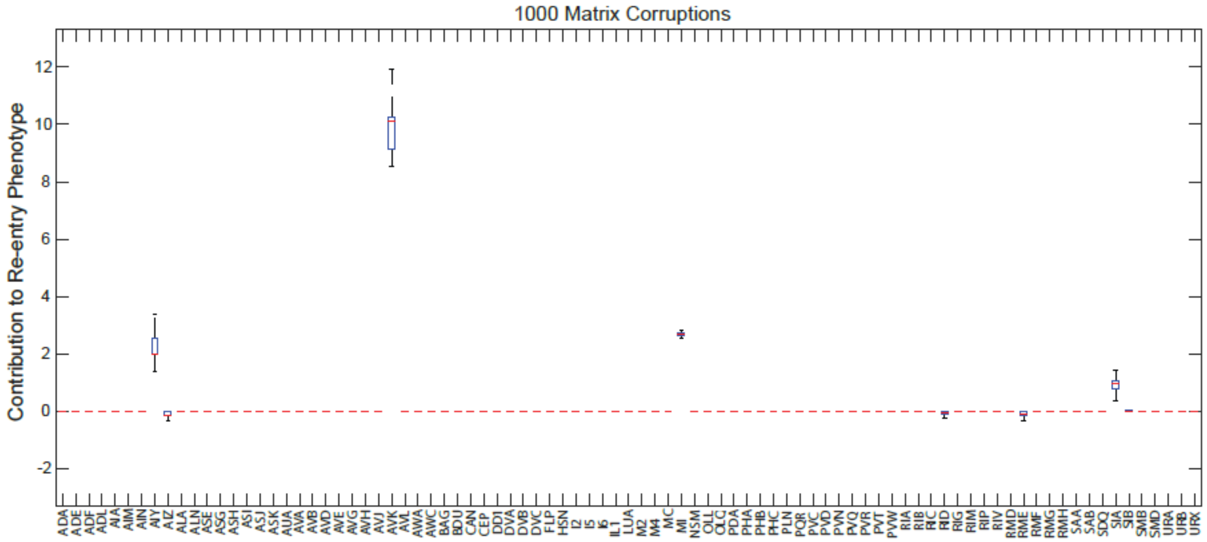

Fig.S3. Validation of compressed sensing solutions via to Arch expression efficiency through measurement matrix corruption

Solutions obtained following perturbing the measurement matrix to mimic differences in expression of Archaerhodopsin (see methods).

bioRxiv preprint doi: https://doi.org/10.1101/2024.04.10.588911; this version posted October 17, 2024. The copyright holder for this preprint (which was not certified by peer review) is the author/funder, who has granted bioRxiv a license to display the preprint in perpetuity. It is made available under aCC-BY 4.0 International license.

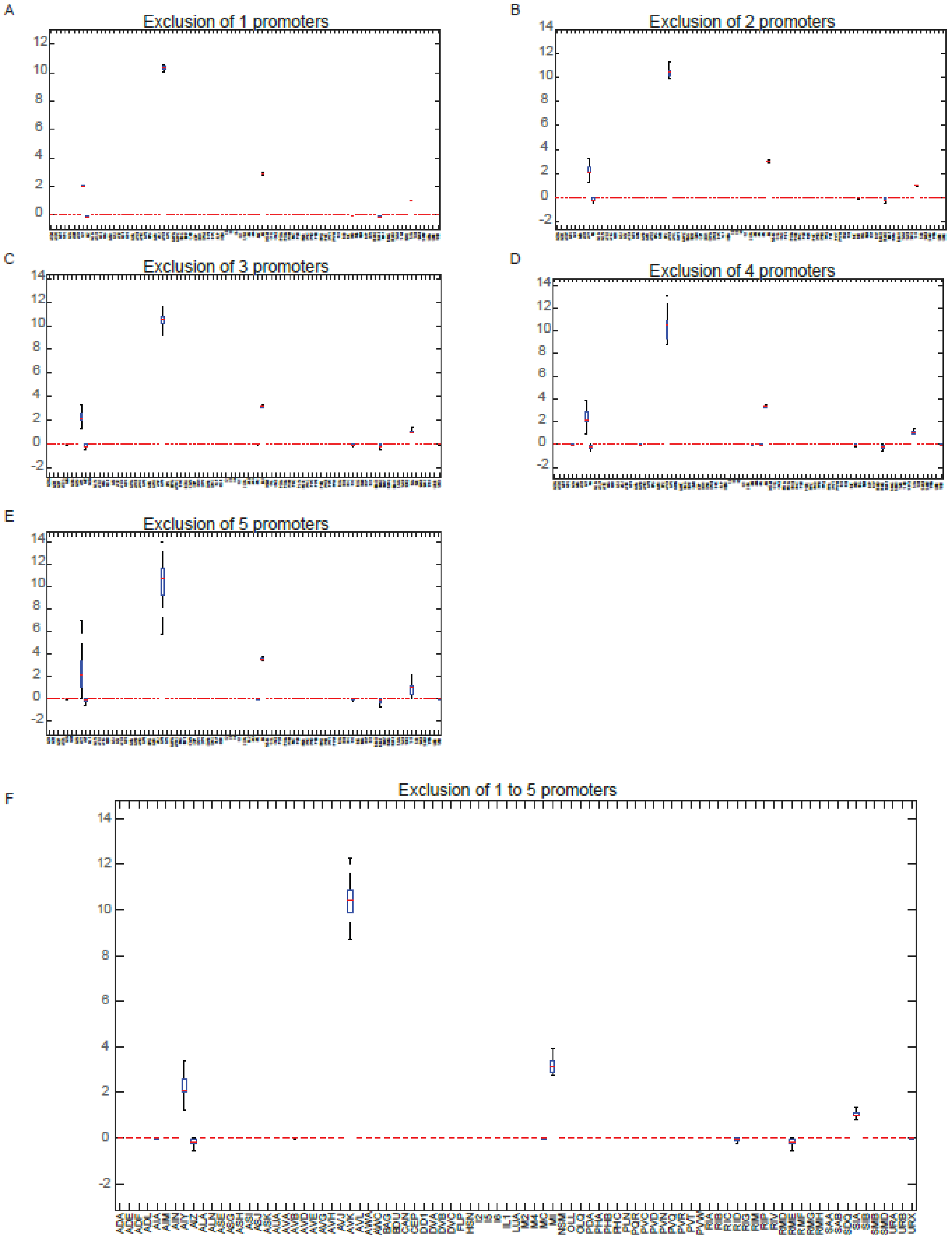

Fig.S4. Validation of robustness of compressed sensing solutions to choice of measurements through promoter removal

To validate that solutions to lawn entry were robust to choice of measurements, 1 (**A**) to 5 (**E**) promoters were removed at random from the measurement matrix (see methods) and solutions were evaluated. Our results showed that our solutions were robust to such removals. (**F**) Aggregated solutions across 1-5 to removals.

bioRxiv preprint doi: https://doi.org/10.1101/2024.04.10.588911; this version posted October 17, 2024. The copyright holder for this preprint (which was not certified by peer review) is the author/funder, who has granted bioRxiv a license to display the preprint in perpetuity. It is made available under aCC-BY 4.0 International license.

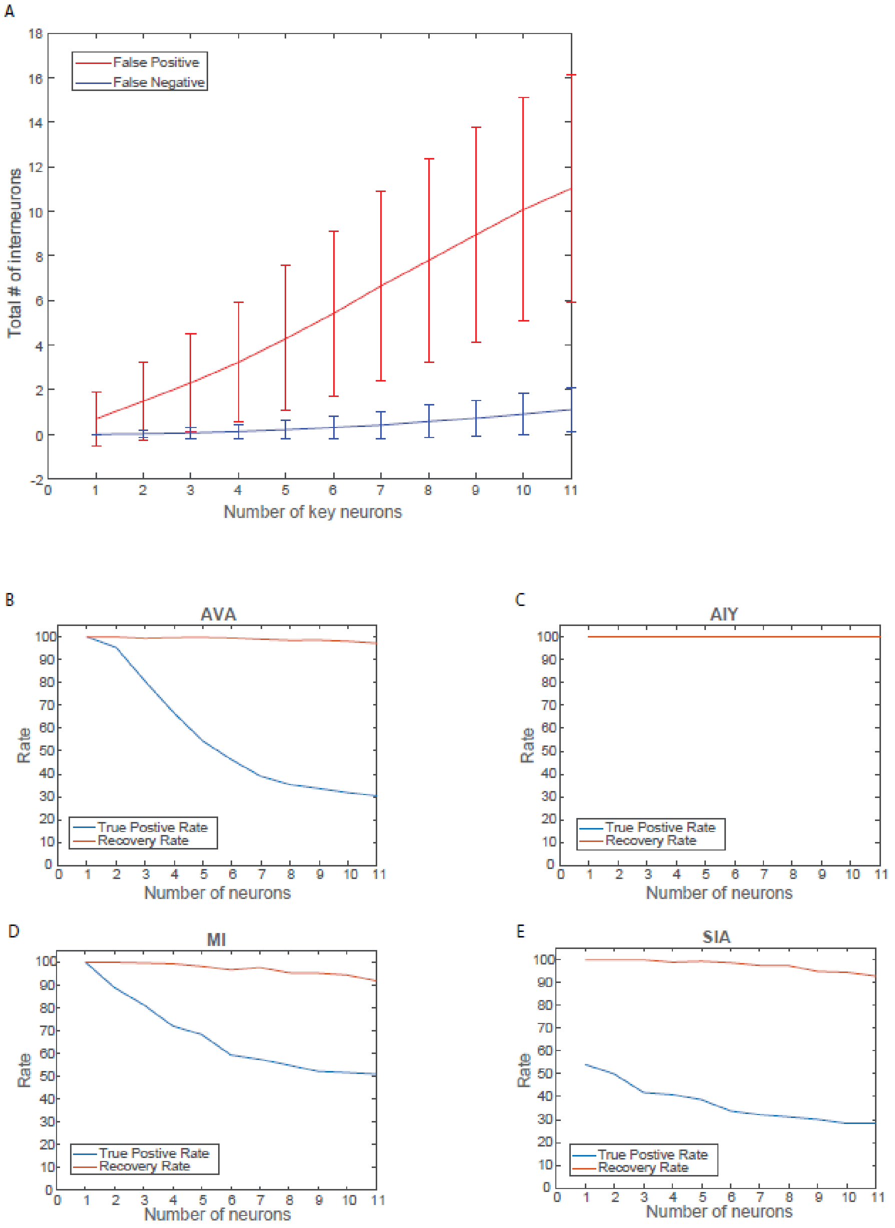

Fig.S5. Recovery and false positive rate for measurement matrix of choice(**A**) False positive and negative rate for recovery of measurement matrix for a randomly simulated set of key neurons (see methods). False positive and recovery rates for each key neuron identified as being important in lawn entry behavior: AVK (**B**), AIY (**C**), MI (**D**) and SIA (**E**).

bioRxiv preprint doi: https://doi.org/10.1101/2024.04.10.588911; this version posted October 17, 2024. The copyright holder for this preprint (which was not certified by peer review) is the author/funder, who has granted bioRxiv a license to display the preprint in perpetuity. It is made available under aCC-BY 4.0 International license.

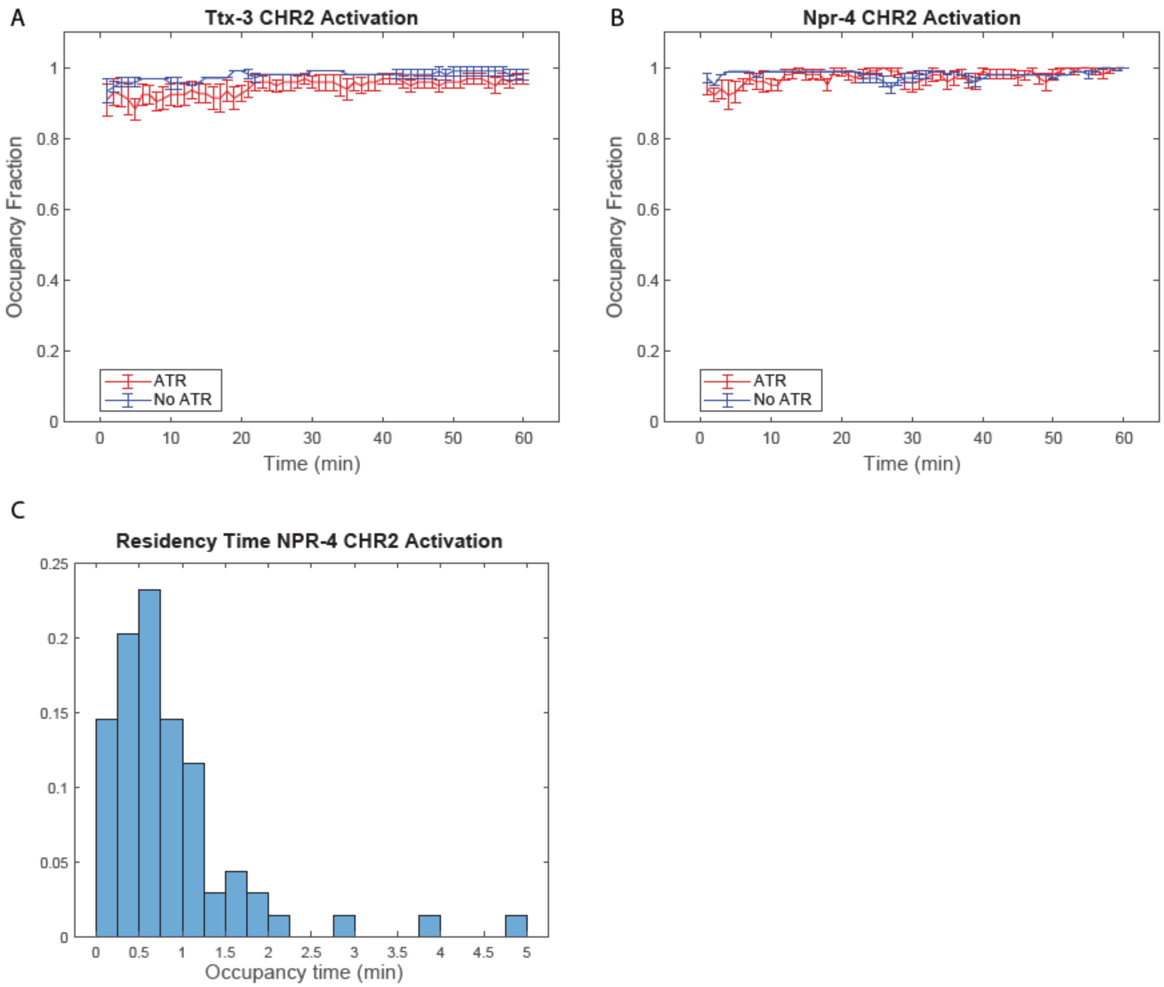

Fig. S6. Activation of re-entry neurons fails to impact lawn occupancy

Activation of AIY **(A)** and Npr-4 expressing neurons **(B)** fails to significantly increase lawn occupancy in evacuated colonies. **(C)** Worms that re-enter lawn after Npr-4 neural activation show very low residency time on PA14 lawns.

bioRxiv preprint doi: https://doi.org/10.1101/2024.04.10.588911; this version posted October 17, 2024. The copyright holder for this preprint (which was not certified by peer review) is the author/funder, who has granted bioRxiv a license to display the preprint in perpetuity. It is made available under aCC-BY 4.0 International license.

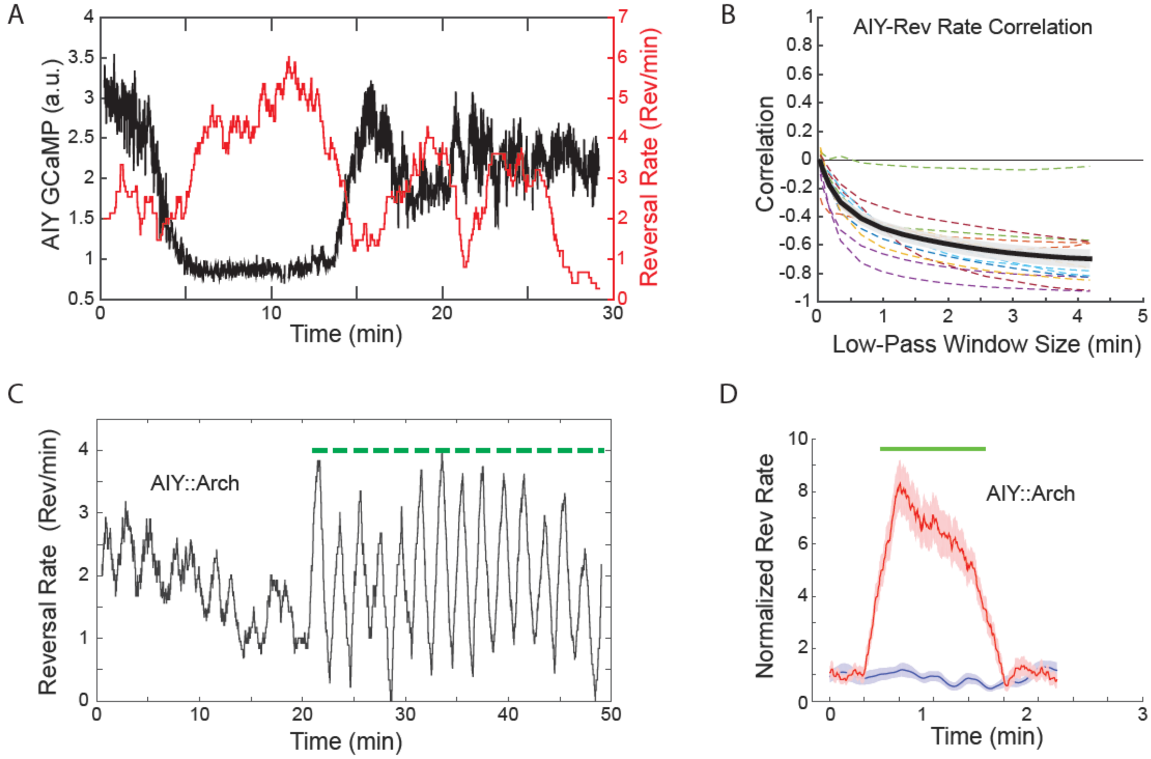

Fig S7. AIY neural activity controls reversal rates.
(A) Sample time series of AIY neural activity vs reversal rate over a 30minute period. AIY neural activity increases with decrease in reversal rate. **(B).** AIY neural activity anticorrelates with reversal rate (n = 13 samples). **(C)** Time series of reversal rate with and without neural inhibition of AIY during navigation on an empty agar plate **(D).** Aggregated reversal data with and without neural inhibition shows an increase in reversal rate with neural inhibition

bioRxiv preprint doi: https://doi.org/10.1101/2024.04.10.588911; this version posted October 17, 2024. The copyright holder for this preprint (which was not certified by peer review) is the author/funder, who has granted bioRxiv a license to display the preprint in perpetuity. It is made available under aCC-BY 4.0 International license.

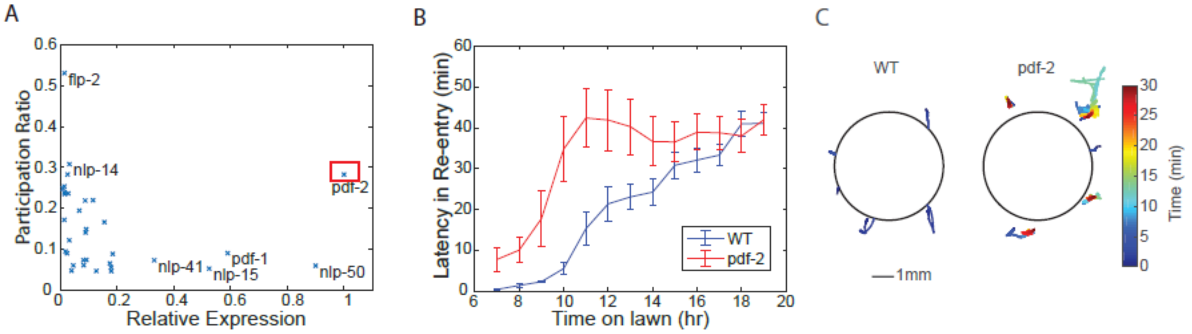

Fig S8. Candidate neuropeptide identification from re-entry neurons
(A) Scatterplot of neuropeptides plotted on an axis of maximum relative expression in one of the two candidate neurons (AIY and SIA) versus the specificity of their expression (as measured by calculated participation ratio, see methods). *pdf-2* is highlighted as a neuropeptide of interest as it showed both high expression and high specificity. **(B)** Plot of worm latency of re-entry onto lawns as a function of time spent on PA14 lawn for loss of function mutant of the neuropeptide *pdf-2* (red) and wild type worms (blue). Mutant worms showed systematic increases in latency of re-entry compared to wild type control. **(C)** Representative center of mass trajectories of worms at the edge of the PA14 lawn taken from 12 to 14hours of PA14 exposure for wild type worms (left) and PDF-2 mutant worms (right) color coded by time

bioRxiv preprint doi: https://doi.org/10.1101/2024.04.10.588911; this version posted October 17, 2024. The copyright holder for this preprint (which was not certified by peer review) is the author/funder, who has granted bioRxiv a license to display the preprint in perpetuity. It is made available under aCC-BY 4.0 International license.

Supplementary Video 1. Time series video of a lawn evacuation assay taken 2hours into the assay

Supplementary Video 2. Time series video of a lawn evacuation assay taken 8hours into the assay

Supplementary Video 3. Time series video of a lawn evacuation assay taken 14hours into the assay.

bioRxiv preprint doi: https://doi.org/10.1101/2024.04.10.588911; this version posted October 17, 2024. The copyright holder for this preprint (which was not certified by peer review) is the author/funder, who has granted bioRxiv a license to display the preprint in perpetuity. It is made available under aCC-BY 4.0 International license.

Supplementary Table 1. Archaerhodopsin lines that constitute the measurement matrix

Promoters	Neurons
wSR853: [pdop-2::Arch-tagRFP]; sraIs[pstr-2::mkO]; lite-1(ce314)x	CEP; SIA; SIB; RIA; RID; ADE
wSR517: [pflp-21::Arch-tagRFP, pBX]; pha-1(e2123)III; lite-1(ce314)x	URX; URA; RMG; MC; M2; AIY
sraEx280[pttx-3::Arch-tagRFP; pBX]; pha-1(e2123)III; lite-1(ce314)x	AIY
wSR230: [pstr-2::Arch-tagRFP; pBX]; pha-1(e2123)III; lite-1(ce314)x	AWC
wSR347: [pflp-3::Arch-GFP; pflp-3::mKO; pBX]; pha-1(e2123)III; lite-1(ce314)x	IL1; OLL; URB; PQR
wSR469: [pflp-19::Arch-tagRFP]; pha-1(e2123)III; lite-1(ce314)x	AWA; URX
wSR495: [pmpz-1prom2::Arch-tagRFP]; pha-1(e2123)III; lite-1(ce314)x	M4; NSM; MC; RMH; RMF; RMD; HSN; SDQ; PVC; PVQ; PVN
wSR545: [pflp-11::Arch-tagRFP, pBX]; pha-1(e2123)III; lite-1(ce314)x	AUA; BAG; DVB; LUA; PHC; PVC; SAB; URX
wSR523: [pflp-12::Arch-tagRFP, pBX]; pha-1(e2123)III; lite-1(ce314)x	BAG; SAA; SMB; AVH; AVJ
wSR496: [pflp-4::Arch-tagRFP]; pha-1(e2123)III; lite-1(ce314)x	NSM; ADL; I5; I6; AWC; FLP
wSR512: [pinx-4:Arch-tagRFP, pBX]; pha-1(e2123)III; lite-1(ce314)x	ADA; ADE; AIN; AUA; AVJ; DVC; FLP; PHA; PHB; PVR; PVT; RIC; RIG; RIM; RIP
wSR500: [pflp-7::Arch-tagRFP]; pha-1(e2123)III; lite-1(ce314)x	ALA; AVG; PHB; PDA; PVW; RIC; SAA
wSR683: [pmod-1::Arch-tagRFP, pBX]; sraIs467[pstr-2::mKO] III; lite-1(ce314)x; pha-1(e2123) III	RID; RME; AIZ; AIY; DD1
wSR448: [pmbr-1::Arch-tagRFP]; pha-1(e2123)III; lite-1(ce314)x	AWC; AIM; RIC; AIN
wSR274: [pser-2prom2::Arch-tagRFP; pBX]; pha-1(e2123)III; lite-1(ce314)x	RME; AIZ; RID; AIY; BDU
wSR454: [plin-11::Arch-tagRFP]; pha-1(e2123)III; lite-1(ce314)x	ADF; ADL; AIZ; RIC; AVG; AVH; AVJ
wSR186: [psra-11::Arch-GFP, psra-11::mKO]; lite-1(ce304) x	AIY; AVB; AIA
wSR453: [pmgl-1::Arch-tagRFP]; pha-1(e2123)III; lite-1(ce314)x	AIA; RMD; NSM
wSR345: [popt-3::Arch-GFP; popt-3::mKO; pBX]; pha-1(e2123)III; lite-1(ce314)x	DVA; AVE; ASJ; OLQ; AIM; CAN
wSR507: [pnmr-1:Arch-tagRFP]; pha-1(e2123)III; lite-1(ce314)x	AVA; AVD; AVE; RIM; AVG
wSR241: [prig-5::Arch-tagRFP; pBX]; pha-1(e2123)III; lite-1(ce314)x	RMD; SMD; I2; MC; M4
wSR352: [pnpr-4::Arch-GFP; pnpr-4::mKO; pBX]; pha-1(e2123)III; lite-1(ce314)x	SIA; SIB; RIC; AVA; RMD; AIY; AVK; BAG
wSR550: [pser-2prom3::Arch-tagRFP, pBX]; pha-1(e2123)III; lite-1(ce314)x	OLL; PVD
wSR224: [podr-2(16)::Arch-tagRFP; pBX]; pha-1(e2123)III; lite-1(ce314)x	SMD; RME
wSR486: [psams-5::Arch-tagRFP];pha-1(e2123)III; lite-1(ce314)x	MI; PVQ

bioRxiv preprint doi: https://doi.org/10.1101/2024.04.10.588911; this version posted October 17, 2024. The copyright holder for this preprint (which was not certified by peer review) is the author/funder, who has granted bioRxiv a license to display the preprint in perpetuity. It is made available under aCC-BY 4.0 International license.

wSR468: [pgpa-14::Arch-tagRFP; pbx]; pha-1(e2123)III; lite-1(ce314)x	ASI; ASJ; ASH; ASK; ADE; PHA; PHB; ALA; AVA; CAN; DVA; PVQ; RIA
wSR288: [podr-2(18)::Arch-tagRFP; pBX]; pha-1(e2123)III; lite-1(ce314)x	SMB; RME; ALN; PLN; RIG
wSR535: [pmgl-3:Arch-tagRFP, pBX]; pha-1(e2123)III; lite-1(ce314)x	NSM; ADF; ASE; AWC; RIB; RIC; BAG
wSR499: [pflp-22::Arch-tagRFP]; pha-1(e2123)III; lite-1(ce314)x; line-3	AIM; ASG; AVA; AVG; AVL; CEP; PVD; PVW; RIC; AIZ; RIV; SMD; URA

bioRxiv preprint doi: https://doi.org/10.1101/2024.04.10.588911; this version posted October 17, 2024. The copyright holder for this preprint (which was not certified by peer review) is the author/funder, who has granted bioRxiv a license to display the preprint in perpetuity. It is made available under aCC-BY 4.0 International license.

Supplementary Table 2. Channelrhodopsin and Halo lines

Promoters	Neurons
wSR281: [pttx-3::ChR2-tagRFP; pBX]; pha-1(e2123); lite-1(ce314)x	AIY
wSR491:[pnpr-4::ChR2-tagRFP]; pha-1(e2123)III; lite-1(ce314)x	SIA; SIB; RIC; AVA; RMD; AIY; AVK; BAG
ZX888: zxIs16 [pflp-1::NpHR::eCFP; lin-15+]	AVK

bioRxiv preprint doi: https://doi.org/10.1101/2024.04.10.588911; this version posted October 17, 2024. The copyright holder for this preprint (which was not certified by peer review) is the author/funder, who has granted bioRxiv a license to display the preprint in perpetuity. It is made available under aCC-BY 4.0 International license.

Supplementary Table 3. GCaMP Lines

Promoters	Neurons Imaged
wSR762: pnpr-4::GCaMP6s; pstr-2::mKO; lite-1(ce314)x	AVK,SIA
wSR490: sraEx490[pttx-3::GCaMP6s(mammalian)]; sraIs467[pstr-2::mKO]; lite-1(ce314)	AIY
